# Analysis of preoperative risk factors for early recurrence after curative pancreatoduodenectomy for resectable pancreatic adenocarcinoma

**DOI:** 10.1515/iss-2021-0034

**Published:** 2022-06-28

**Authors:** Pipit Burasakarn, Anuparp Thienhiran, Pusit Fuengfoo, Sermsak Hongjinda

**Affiliations:** Division of HPB Surgery, Department of Surgery, Phramongkutklao Hospital, Bangkok, Thailand

**Keywords:** disease-free survival, overall survival, pancreatic ductal adenocarcinoma, pancreatoduodenectomy, recurrence

## Abstract

**Objectives:**

To investigate the risk factors for early recurrence after curative pancreatoduodenectomy for resectable pancreatic ductal adenocarcinoma.

**Methods:**

All data were retrospectively collected from patients with resectable pancreatic ductal adenocarcinoma who had undergone pancreatoduodenectomy at the Department of Surgery, Phramongkutklao Hospital, from January 2015 to December 2020. The preoperative and perioperative risk factors were included into the analysis.

**Results:**

In total, 34 patients were included in the study. The median time for recurrence and median survival time were 17 and 20 months, respectively. The 1, 3, and 5 year disease-free survival rates were 59.6%, 23.87%, and 23.87%, respectively, while the 1, 3, and 5 year overall survival rates were 81%, 24.7%, and 12.4%, respectively. Seventeen patients (50%) from a total of 34 patients had recurrence, and ten patients (29.41%) had recurrence within 12 months. The independent preoperative risk factor associated with adverse disease-free survival was tumor size > 4 cm (hazard ratio [HR], 14.34, p=0.022). The perioperative risk factors associated with adverse disease-free survival were pathological lymphovascular invasion (HR, 4.31; p=0.048) and non-hepatopancreatobiliary surgeon (HR, 5.9; p=0.022). Risk factors associated with poor overall survival were microscopical margin positive (R1) resection (HR, 3.68; p=0.019) and non-hepatopancreatobiliary surgeon (HR, 3.45; p=0.031).

**Conclusions:**

Tumor size > 4 cm from the preoperative imaging study was a poor prognostic factor for early recurrence after curative pancreatoduodenectomy for resectable pancreatic adenocarcinoma indicated that they may have radiological occult metastasis, thus, staging laparoscopy may reduce the number of unnecessary laparotomies and avoid missing radiologically negative metastases.

## Introduction

Pancreatic ductal adenocarcinoma (PDAC) has a dismal prognosis, with a reported five-year survival rate of 8.5%. PDAC remains the most lethal cancer [1]. Patients with PDAC are usually asymptomatic until they become advanced at the time of diagnosis. Surgery is the only curative treatment, and provides long-term survival; however, only 20% of all patients with PDAC are suitable for surgical resection. Moreover, early recurrence after resection is a problem, with a reported median time to recurrence of 10–12 months [2, 3]. In the era of modern chemotherapy, neoadjuvant chemotherapy can downstage the cancer and is widely used for borderline resectable PDAC. However, neoadjuvant chemotherapy may be applied to patients with resectable PDAC that have the potential for early recurrence for the following purposes: (1) to increase the proportion of patients who receive chemotherapy, (2) help select suitable patients for major operations with high morbidity, and (3) help eliminate distant occult metastasis since resectable PDAC may have metastasis at the time of diagnosis or on surgical exploration [4, 5].

This study aimed to find the preoperative risk factors associated with early recurrence after surgery for resectable PDAC according to the National Comprehensive Cancer Network (NCCN) version 2.2021 [6].

## Materials and methods

### Patient characteristics and study design

This study was approved by the ethical review board of Phramongkutklao Hospital, Bangkok, Thailand. Written informed consent from each patient was waived owing to the retrospective design of the study. All data were retrospectively collected from patients with resectable PDAC who had undergone pancreatoduodenectomy at the Department of Surgery, Phramongkutklao Hospital, between January 2015 and December 2020. The inclusion criteria were as follows: (1) patients diagnosed with PDAC confirmed by a diagnostic radiologist or pathologist in cases that underwent endoscopic ultrasonography-guided biopsy, (2) resectable cases (no tumour contact to celiac axis, superior mesenteric artery, and common hepatic artery, or no tumor contact of the superior mesenteric vein or portal vein, or ≤ 180° contact without vein contour irregularity) [6]. The exclusion criteria were as follows: (1) patients with distant metastasis (confirmed by gross or microscopic pathology intra-operatively), and (2) palliative resection cases. In total, 34 patients were included in the study.

The primary outcome of this study was the preoperative risk factors associated with poor disease-free survival (DFS), followings risk factors were included into analysis; age, gender, size of tumor, positive lymph node from imaging, serum albumin level, serum bilirubin level, serum carcinoembryonic antigen (CEA) level and serum carbohydrate antigen (CA19-9) level. The secondary outcome was the perioperative prognostic factors associated with overall survival (OS), followings prognostic factors were included into analysis; age, gender, size of tumour, positive lymph node from imaging, serum albumin level, serum bilirubin level, serum carcinoembryonic antigen (CEA) level, serum carbohydrate antigen (CA19-9) level, type of preoperative biliary drainage, length of hospital stay, operative time, type of operated surgeon, intraoperative blood loss, postoperative complications, resection status (R), tumour differentiation, lymphovascular invasion, perineural invasion, T-staging, N-staging, and adjuvant chemotherapy.

### Definitions

Preoperative imaging and laboratory tests were performed within 30 days before surgery, and serum carbohydrate antigen 19–9 (CA 19–9) was collected after biliary drainage in jaundiced patients. Morbidity was classified using the Clavien–Demartines–Dindo system [7]. Postoperative pancreatic fistula (POPF) [8], hemorrhage (PPH) [9], and delayed gastric emptying time (DGE) [10] were defined according to the International Study Group criteria. Perioperative mortality was defined as death within 90 days of surgery from any cause.

### Surgical procedures

All patients underwent preoperative evaluation for disease staging using contrast-enhanced abdominal multidetector computed tomography and/or magnetic resonance imaging with cholangiopancreatography. Preoperative biliary drainage was routinely performed for patients with cholangitis, including endoscopic biliary stent drainage, percutaneous transhepatic biliary drainage, or a combination of both. Conventional pancreatoduodenectomy with regional lymphadenectomy was performed in all the cases, and child’s reconstruction method was used [11]. All patients underwent duct-to-mucosa pancreatojejunostomy using an internal or external stent insertion depending on the surgeon’s preference, and surgical drainage was performed in each patient.

### Postoperative management and follow-up

Gemcitabine, capecitabine, or fluorouracil plus leucovorin have been used as adjuvant chemotherapy agents for pancreatic adenocarcinoma in our institution and were considered for patients with lymph node metastasis or microscopic positive margin resection (R1) based on the surgeon and medical oncologist’s judgement within the first 12 weeks postoperatively. The patients were monitored every 3 to 6 months for the first 24 months, and then every 6 to 12 months as clinically indicated. Recurrence was determined using blood tests and tumor markers (carcinoembryonic antigen and CA 19–9) at every follow-up examination and computed tomography (CT) examinations performed at the time of follow-up.

### Statistics analyses

Continuous variables were compared using the Student’s t-test or the nonparametric Mann–Whitney test, as appropriate, and are presented as mean ± standard deviation and median (interquartile range) for non-normally distributed data. Categorical variables were compared using the chi-square test or Fisher’s exact test, as appropriate, and are presented as numbers (percentages). OS was calculated from the date of surgery to the date of death. Disease-free survival (DFS) was calculated from the date of surgery to the date of the first recurrence at any site. Survival rates were calculated using the Kaplan–Meier method and compared using log-rank tests. The Cox proportional hazards model was used to calculate the hazard ratio (HR) with 95% confidence interval (CI) for the risk factors for disease-free and overall survival rates. All statistical analyses were performed using STATA/IC 14.0 (StataCorp. 2015. Stata Statistical Software: Release 14. College Station, TX, StataCorp LP, USA). Statistical significance was set at p<0.05. The data of patients who were lost to follow-up or quit were treated as censored.

## Results

Patient characteristics and demographic data, as well as perioperative data, are shown in Table 1. The mean age was 66.3, and the majority were male. The median preoperative CA19-9 was 114 In total, 60.6% of patients underwent preoperative biliary drainage divided into percutaneous transhepatic biliary drainage (21.21%) and endoscopic stent drainage (39.9%), with a median serum total bilirubin of 9.01 (2.6, 16.2). Combined portal vein resection was performed in 6 patients (18.2%).

**Table 1:** Patient demographics, characteristics, and perioperative data.

Age, mean ± SD	66.30 ± 10.95
Male gender	18 (54.5%)
Size of tumor from imaging, mean ± SD	2.82 ± 1.38
Serum albumin (g/dL), mean ± SD	3.59 ± 0.54
Serum bilirubin (mg/dL), median (IQR)	9.01 (2.60, 16.2)
Preop CEA, median (IQR)	4.45 (2.73, 5.99)
Preop CA 19–9, median (IQR)	114 (28.18, 797)
Preoperative biliary drainage	20 (60.6%)
Percutaneous transhepatic drainage/endoscopic stent drainage	7 (21.2%)/13 (39.4%)
Body mass index, median (IQR)	21.22 (19.65, 24.24)
Hospital stay, median (IQR)	12 (10, 28)
Operative time, mean ± SD	529.39 ± 146.66
Combine portal vein resection	6 (18.2%)
Blood loss, mean ± SD	1246.97 ± 1432.27
Overall morbidity/major morbidity (CD grade (III/IV))	15 (45.45%)/6 (18.18%)
Postoperative pancreatic fistula	7 (21.2%)
Post pancreatectomy hemorrhage	3 (9.1%)
Biliary-enteric anastomosis leakage	2 (6.1%)
Delayed gastric emptying	2 (6.1%)
Wound complications	3 (9.1%)
Microscopic positive margin (R1) resection	8 (24.2%)
Tumor differentiation (well/moderately/poorly)	11 (40.7%)/14 (51.8%)/2 (7.4%)
Size of tumor from pathology, mean ± SD	3.23 ± 1.40
Lymphovascular invasion	20 (60.6%)
Perineural invasion	27 (84.4%)
T stage; T1/T2/T3	6 (18.2%)/18 (54.6%)/8 (24.2%)
N stage; N0/N1/N2	17 (51.5%)/12 (36.4%)/4 (12.1%)
Adjuvant chemotherapy	21 (63.6%)
Adjuvant radiotherapy	6 (18.2%)

SD, standard deviation; IQR, interquartile range; R1, microscopic positive margin status; CD, grade III/IV major morbidity according to Clavien–Demartines–Dindo system; CEA, carcinoembryonic antigen; CA19-9, Carbohydrate antigen 19-9.

### Perioperative outcomes

The mean operative time was 529.4 min with a mean blood loss of 1,247 mL. Overall morbidity was 45.5%, this included major morbidity (Clavein-Dindo III/IV) in 6 patients (18.2%), POPF in 7 patients (21.2%), PPH in 3 patients (9%), DGE in 1 patient (3.05%), and biliary-enteric leakage in one patient (3.05%). The median length of hospital stay was 12 days, and in-hospital deaths occurred in two patients resulting from early postoperative hemorrhage.

### Long-term outcomes

**Figure 1:**
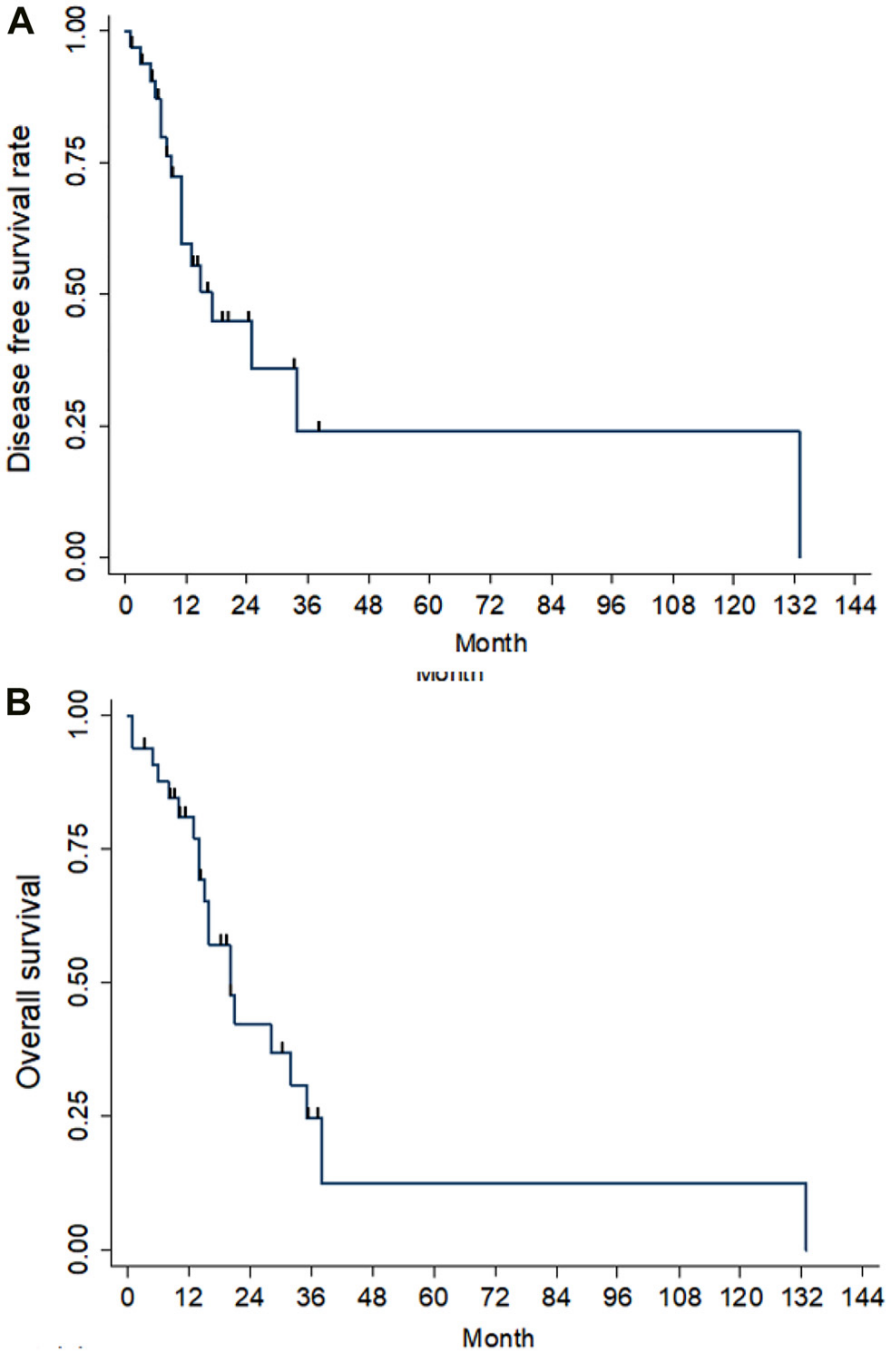
The Kaplan–Meier survival curves of the patients in this study. (A) disease-free survival (B) overall survival.

**Figure 2:**
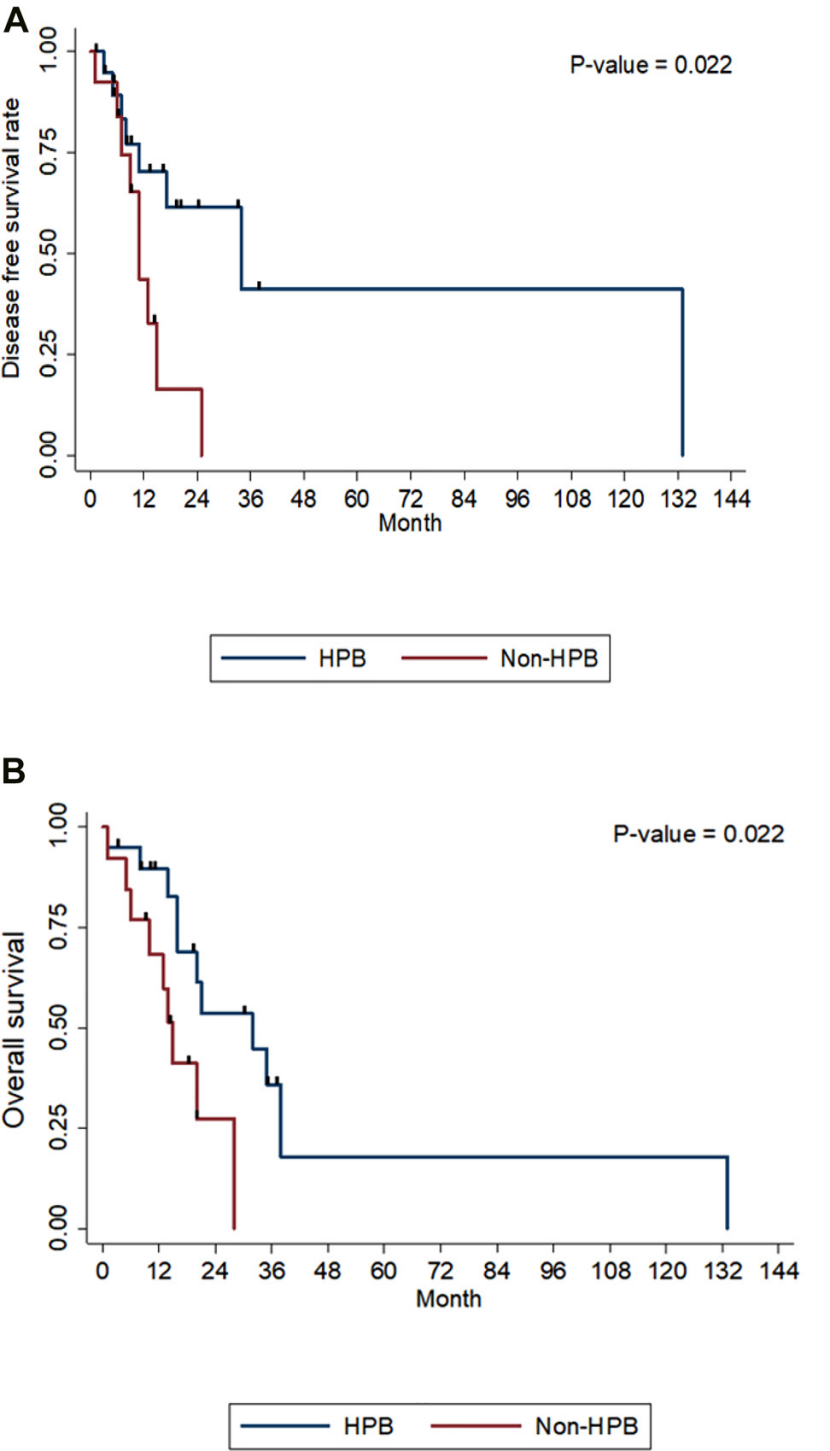
The Kaplan–Meier survival curves of the hepatopancreatobiliary (HPB) surgeon and non-HPB surgeon groups. (A) disease-free survival (B) overall survival.

Recurrence patterns are shown in Table 3. Ten out of the 34 patients included had recurrence within 1 year. The median time to recurrence and median survival time were 17 and 20 months, respectively. The 1, 3, and 5 year disease-free survival rates were 59.6%, 23.87%, and 23.87%, respectively, while the 1, 3, and 5 year OS rates were 81%, 24.7%, and 12.4%, respectively (Figure 1). Univariate and multivariate hazard ratio analyses of risk factors for DFS and OS are summarized in Table 2; the only significant preoperative risk factor associated with adverse DFS was tumor size > 4 cm (HR, 14.34, p=0.022); however, the perioperative risk factors associated with adverse DFS were pathological lymphovascular invasion (HR, 4.31, p=0.048) and non-hepatopancreatobiliary surgery (HR, 5.9, p=0.022). The perioperative risk factors associated with poor OS were microscopical margin positive (R1) resection (HR, 3.68, p=0.019) and non-hepatopancreatobiliary surgery (HR, 3.45, p=0.031) (Figure 2).

**Table 2:** Cox’s proportional-hazard analysis of the risk factors for adverse disease-free survival and overall survival outcomes.

Factors	Disease-free survival				Overall survival			
	Univariate		Multivariate		Univariate		Multivariate	
	HR (95% CI)	p-Value	Adjusted HR (95% CI)	p-Value	HR (95% CI)	p-Value	Adjusted HR (95% CI)	p-Value
Male (vs. female)	1.33 (0.48–3.68)	0.582			2.05 (0.74–5.70)	0.170		
Preop CA 19–9 ≥ 37 (vs. < 37)	1.67 (0.48–5.89)	0.422			0.86 (0.30–2.45)	0.784		
Size of tumor ≥ 4 cm (vs. < 4 cm)	1.21 (0.28–5.15)	0.801	14.34 (1.48–139.07)	0.022	0.5 (0.13–1.89)	0.304		
Preoperative biliary drainage (vs. no drainage)	1.71 (0.61–4.83)	0.311			1.14 (0.45–2.93)	0.778		
Non-HPB surgeon (vs. HPB surgeon)	3.20 (1.11–9.29)	0.032	5.90 (1.29–27.09)	0.022	3.07 (1.11–8.49)	0.031	3.45 (1.12–10.64)	0.031
Postoperative morbidity (vs. no morbidity)	1.32 (0.49–3.57)	0.578			1.43 (0.58–3.54)	0.438		
POPF (vs. no POPF)	2.68 (0.83–8.62)	0.098			2.64 (0.99–7.08)	0.054		
PPH (vs. no PPH)	2.16 (0.47–9.88)	0.323			3.33 (0.93–11.89)	0.064		
R1 resection status (vs. R0)	1.90 (0.58–6.21)	0.291			2.63 (0.97–7.12)	0.057	3.68 (1.23–11.00)	0.019
Lymphovascular invasion (vs. no invasion)	2.14 (0.69–6.66)	0.190	4.31 (1.01–18.32)	0.048	1.29 (0.49–3.41)	0.607		
N1 stage (vs. N0)	0.91 (0.32–2.58)	0.864			0.68 (0.25–1.86)	0.453		
N2 stage (vs. N0)	1.36 (0.16–11.59)	0.780			1.63 (0.34–7.76)	0.542		

R0, microscopic negative margin; R1, microscopic positive margin; HR, hazard ratio; CI, confident interval; POPF, postoperative pancreatic fistula; PPH, post pancreatectomy hemorrhage; CA19-9, Carbohydrate antigen 19–9; HPB, hepato-pancreato-biliary.

## Discussion

Although curative surgery remains the mainstay treatment for pancreatic adenocarcinoma, early recurrence occurs frequently, with up to 80% of patients experiencing a short disease-free interval [12, 13], leading to poor prognosis. These findings suggest that even resectable pancreatic adenocarcinoma from radiological studies still has the potential to have occult metastases. Adjuvant treatment including chemotherapy, radiotherapy, or a combination of both was applied to improve disease-free and overall survivals after surgery with favorable results compared to surgery alone [14], [15], [16]; however, 1/3 of patients were unable to receive adjuvant treatment due to postoperative complications [17]. Therefore, neoadjuvant chemotherapy cancer not only for borderline resectable cases [18, 19] but also in resectable pancreatic; conversely, randomised trials are required to confirm the true benefit of neoadjuvant chemoradiotherapy (Table 3).

**Table 3:** Recurrence pattern after curative pancreatoduodenectomy for resectable pancreatic adenocarcinoma (total 17 patients).

Recurrence patterns	0–12 months	12–24 months	24–48 months
Local only	3 (17.6%)	–	2 (11.8%)
Regional lymph node only	1 (5.9%)	3 (17.6%)	–
Liver only	1 (5.9%)	–	–
Peritoneal carcinomatosis	4 (23.5%)	–	–
Multiple sites (include lung metastasis)	1 (5.9%)	–	2 (11.8%)
Total	10 (58.8%)	3 (17.6%)	4 (23.5%)

Nevertheless, not all patients with resectable PDAC will benefit from neoadjuvant treatment; only patients with potential for early recurrence may benefit from this treatment. Our results indicated that tumor size > 4 cm on cross-sectional imaging (HR, 14.34, p=0.022) was the only significant poor prognostic factor for early recurrence, while other preoperative risk factors such as positive lymph nodes from imaging or elevated CA 19–9 were considered to be not statistically significant. Indicated that patients with resectable PDAC size more than 4 cm may have radiological occult metastasis [29], thus, staging laparoscopy may reduce the number of unnecessary laparotomies and avoid missing radiologically negative metastases [30] or even they may candidates for neoadjuvant chemotherapy [31, 32]. Some retrospective studies aimed to identify the poor prognostic factors associated with early recurrence after curative resection, and Nishio et al. reported that 32 out of 90 patients had recurrence within 1 year after curative surgery, in which preoperative serum CA19-9 level > 529 U/ml was an independent predictor of recurrence within 1 year [20]. Izumo et al. reported that the preoperative independent risk factors for early recurrence and poor outcomes for resectable pancreatic cancer were CA 19–9 > 37 U/mL and tumor size >2.6 cm [21]. Other relevant prognostic factors includes tumour size [22, 23], lymph node metastases/lymphovascular invasion [24, 25], and resection margin status [26], which is consistent with our results. High-volume centers or experienced surgeons should be consulted to improve prognosis after surgery from high morbidity operation [27, 28]. Our results also suggest that pancreatoduodenectomy should be performed by experienced hepatobiliary surgeons to achieve long-term survival.

The main limitation of this study was its retrospective design, which was performed in a single institution with a low volume of patients with resectable PDAC, the restricted number of patients may have an impact on the multivariate analysis. Further well design, randomized studies are required.

## Conclusions

Tumor size >4 cm from the preoperative imaging study was a poor prognostic factor for early recurrence after curative pancreatoduodenectomy for resectable pancreatic adenocarcinoma indicated that they may have radiological occult metastasis, thus, staging laparoscopy may reduce the number of unnecessary laparotomies and avoid missing radiologically negative metastases.

## Supplementary Material

Supplementary Material
