# Supplementary material for: Analysis of preoperative risk factors for early recurrence after curative pancreatoduodenectomy for resectable pancreatic adenocarcinoma
Source: Innov Surg Sci. 2022 Jun 28;7(1):5–11. doi: 10.1515/iss-2021-0034 (PMC9352185; doi:10.1515/iss-2021-0034)
Supplement: Supplementary file 1 — Supplementary Material [file j_iss-2021-0034_suppl.pdf]

## Reviewer Assessment

# P. Burasakarn et al.: Analysis of preoperative risk factors for early recurrence after curative pancreatoduodenectomy for resectable pancreatic adenocarcinoma

## Reviewers' Comments to Original Submission

**Reviewer 1: anonymous**

Date received: 26-Oct-2021
Reviewer recommendation: **Return to author for major modifications**
Reviewer overall scoring: **Low**

Assessment Form scores: 5 = High/Yes; 3 = Medium/Adequate; 1 = Low

| | | | | | |
|---|---|---|---|---|---|
| Is the subject area appropriate for the journal | 5 | | | | |
| Does the title clearly reflect the paper's content? | | | 3 | | |
| Does the abstract clearly reflect the paper's content | | | 3 | | |
| Do the keywords clearly reflect the paper's content? | 5 | | | | |
| Does the introduction present the problem clearly? | | 4 | | | |
| Are the results/ conclusions justified? | | | | 2 | |
| How comprehensive and up-to-date is the subject matter presented? | | | | 2 | |
| How adequate is the data presentation? | | | | 2 | |
| Are units and terminology used correctly? | 5 | | | | |
| Is the number of cases adequate? | | | | 2 | |
| Are the experimental methods/ clinical studies adequate? | | | | 2 | |
| Is the length appropriate in relation to the content? | | 4 | | | |
| Does the reader get new insights from the article? | | | | 2 | |
| Please rate the practical significance. | | | | 2 | |
| Please rate the accuracy of methods. | | | | 2 | |
| Please rate the statistical evaluation and quality control. | | | | 2 | |
| Please rate the appropriateness of the figures and tables. | | | 3 | | |
| Please rate the appropriateness of the references. | | | 3 | | |
| Please evaluate the writing style and use of language. | | | 3 | | |
| Please judge the overall scientific quality of the manuscript. | | | | 2 | |
| Are the methods used worthy of reproduction in greater deal? | No | | | | |
| Would you be willing to review a revision of this manuscript? | Yes | | | | |

**Comments to author:** A retrospective study is presented investigating risk factors for early recurrence in in patients under going partial pancreatoduodenectomy in curative attempt. The rationale to perform this study is given, the methods and reporting need improvement.
I have a some concerns to be addressed:
Abstract:
In the methods section the risk factors should be outlined. "34 patients were..." should be part of the results. It should be non-HPB "surgeon" and not "surgery" according to table 2. The conclusion that patients would profit from neoadjuvant therapy is not justified!

Methods: Nowhere in the manuscript the risk factors are outlined and justified. Please tell us why you selected those risk factors. What about malnutrition or or UICC (not just T/N) or other factors? Which other factors did you have in your database? How many patients did complete the adjuvant therapy?
The sample is just too small for a multivariate analysis.
Discussion:
The limitation section should really discuss the influence of the limitations on the results.
The conclusion that patients with 4cm would profit from neodjuvant therapy cannot be made upon your presented data. To make such a statement it would need an RCT or a large retrospective tudy including neodjuvant therapy as a factor. Please revise.
Sample is too small..

## Authors' Response to Reviewer Comments

Date received: 08-Jan-2022

**Response to reviewer 1**

Comments to the Author
A retrospective study is presented investigating risk factors for early recurrence in in patients under going partial pancreatoduodenectomy in curative attempt. The rationale to perform this study is given, the methods and reporting need improvement.
I have a some concerns to be addressed:
Abstract:
1. In the methods section the risk factors should be outlined.
*Response: We would like to thank the Editor for the valuable comment*
*We revised the abstract according to your comment, however, the complete outline of risk factors are in the manuscript part.*
*(Page 1, Line 8) abstract*
*All data were retrospectively collected from patients with resectable pancreatic ductal adenocarcinoma who had undergone pancreatoduodenectomy at the Department of Surgery, Phramongkutklao Hospital, from January 2015 to December 2020. The preoperative and perioperative risk factors were included into the analysis.*
*(Page 3, Line 29 to Page 4 Line 8) methods*
*The primary outcome of this study was the preoperative risk factors associated with poor disease-free survival (DFS), followings risk factors were included into analysis; age, gender, size of tumour, positive lymph node from imaging, serum albumin level, serum bilirubin level, serum Carcinoembryonic Antigen (CEA) level and serum Carbohydrate antigen (CA19-9) level. The secondary outcome was the perioperative prognostic factors associated with overall survival (OS), followings prognostic factors were included into analysis; age, gender, size of tumour, positive lymph node from imaging, serum albumin level, serum bilirubin level, serum Carcinoembryonic Antigen (CEA) level, serum Carbohydrate antigen (CA19-9) level, type of preoperative biliary drainage, length of hospital stay, operative time, type of operated surgeon, intraoperative blood loss, postoperative complications, resection status (R), tumour differentiation, lymphovascular invasion, perineural invasion, T-staging, N-staging, and adjuvant chemotherapy.*

2. "34 patients were..." should be part of the results.

*Response: We would like to thank the Editor for the valuable comment*

*We revised the abstract according to your comment.*

*(Page 1, Line 10)*

*In total, 34 patients were included in the study*

3. It should be non-HPB "surgeon" and not "surgery" according to table 2.

*Response: We would like to thank the Editor for the valuable comment*

*We revised the abstract according to your comment.*

*(Page 1, Line 17-19)*

*non-hepatopancreatobiliary surgeon (HR, 5.9; p=0.022). Risk factors associated with poor overall survival were microscopical margin positive (R1) resection (HR, 3.68; p=0.019) and non-hepatopancreatobiliary surgeon (HR, 3.45; p=0.031).*

4. The conclusion that patients would profit from neoadjuvant therapy is not justified!

*Response: We would like to thank the Editor for the valuable comment*

*We revised the abstract according to your comment.*

*(Page1, Line 21-24)*

*Tumour size > 4 cm from the preoperative imaging study was a poor prognostic factor for early recurrence after curative pancreatoduodenectomy for resectable pancreatic adenocarcinoma indicated that they may have radiological occult metastasis, thus, staging laparoscopy may reduce the number of unnecessary laparotomies and avoid missing radiologically negative metastases.*

Methods:

5. Nowhere in the manuscript the risk factors are outlined and justified. Please tell us why you selected those risk factors.

*Response: We would like to thank the Editor for the valuable comment*

*We selected all possible preoperative risk factors to analysis in this study and outline in the methods section*

*(Page 3, Line 29 to Page 4 Line 8)*

*The primary outcome of this study was the preoperative risk factors associated with poor disease-free survival (DFS), followings risk factors were included into analysis; age, gender, size of tumor, positive lymph node from imaging, serum albumin level, serum bilirubin level, serum Carcinoembryonic Antigen (CEA) level and serum Carbohydrate antigen (CA19-9) level. The secondary outcome was the perioperative prognostic factors associated with overall survival (OS), followings prognostic factors were included into analysis; age, gender, size of tumor, positive lymph node from imaging, serum albumin level, serum bilirubin level, serum Carcinoembryonic Antigen (CEA) level, serum Carbohydrate antigen (CA19-9) level, type of preoperative biliary drainage, length of hospital stay, operative time, type of operated surgeon, intraoperative blood loss, postoperative complications, resection status (R), tumor differentiation, lymphovascular invasion, perineural invasion, T-staging, N-staging, and adjuvant chemotherapy.*

6. What about malnutrition or UICC (not just T/N) or other factors?

*Response: We would like to thank the Editor for the valuable comment*

*Serum albumin level was also included into the risk factors. The other factor was already outline in our manuscript*

*(Page 3, Line 29 to Page 4 Line 8)*

*The primary outcome of this study was the preoperative risk factors associated with poor disease-free survival (DFS), followings risk factors were included into analysis; age, gender, size of tumor, positive lymph node from imaging, serum albumin level, serum bilirubin level, serum Carcinoembryonic Antigen (CEA) level and serum Carbohydrate antigen (CA19-9) level. The secondary outcome was the perioperative prognostic factors associated with overall survival (OS), followings prognostic factors were included into analysis; age, gender, size of tumor, positive lymph node from imaging, serum albumin level, serum bilirubin level, serum Carcinoembryonic Antigen (CEA) level, serum Carbohydrate antigen (CA19-9) level, type of preoperative biliary drainage, length of hospital stay, operative time, type of operated surgeon, intraoperative blood loss, postoperative complications, resection status (R), tumor differentiation, lymphovascular invasion, perineural invasion, T-staging, N-staging, and adjuvant chemotherapy.*

7. Which other factors did you have in your database?
*Response: We would like to thank the Editor for the valuable comment*

*Our database of risk factors that included into analysis were followings, age, gender, size of tumor, positive lymph node from imaging, serum albumin level, serum bilirubin level, serum Carcinoembryonic Antigen (CEA) level, serum Carbohydrate antigen (CA19-9) level, type of preoperative biliary drainage, length of hospital stay, operative time, type of operated surgeon, intraoperative blood loss, postoperative complications, resection status (R), tumor differentiation, lymphovascular invasion, perineural invasion, T-staging, N-staging, and adjuvant chemotherapy*

8. How many patients did complete the adjuvant therapy?
*Response: We would like to thank the Editor for the valuable comment*
*In our study, only 21 patients (63.64%) were received adjuvant chemotherapy and completed the chemotherapy course every patient.*

9. The sample is just too small for a multivariate analysis.
*Response: We would like to thank the Editor for the valuable comment*
*We agreed with you comment and mentioned into the limitation part.*
*(Page 7, Line 10-12)*
*The main limitation of this study was its retrospective design, which was performed in a single institution with a low volume of patients with resectable PDAC, the restricted number of patients may have an impact on the multivariate analysis. Further well design, randomized studies are required.*

Discussion:
10. The limitation section should really discuss the influence of the limitations on the results.
*Response: We would like to thank the Editor for the valuable comment*
*We have revised the limitation part of our manuscript.*
*(Page 7, Line 10-12)*

*The main limitation of this study was its retrospective design, which was performed in a single institution with a low volume of patients with resectable PDAC, the restricted number of patients may have an impact on the multivariate analysis. Further well design, randomized studies are required.*

11. The conclusion that patients with 4cm would profit from neodjuvant therapy cannot be made upon your presented data. To make such a statement it would need an RCT or a large retrospective study including neoadjuvant therapy as a factor. Please revise.
*Response: We would like to thank the Editor for the valuable comment*
*We have revised our manuscript according to your comments.*
*(Page 6, Line 26-29) Discussion*

*Indicated that patients with resectable PDAC size more than 4 cm may have radiological occult metastasis [29], thus, staging laparoscopy may reduce the number of unnecessary laparotomies and avoid missing radiologically negative metastases [30] or even they may candidates for neoadjuvant chemotherapy [31-32].*
*(Page 7, Line 15-18) Conclusion*

*Tumour size > 4 cm from the preoperative imaging study was a poor prognostic factor for early recurrence after curative pancreatoduodenectomy for resectable pancreatic adenocarcinoma indicated that they may have radiological occult metastasis, thus, staging laparoscopy may reduce the number of unnecessary laparotomies and avoid missing* radiologically negative metastases.

12. Sample is too small.
*Response: We would like to thank the Editor for the valuable comment*
*We agree with the comment and revise the limitation part of our manuscript.*
*(Page 7, Line 10-12)*

*The main limitation of this study was its retrospective design, which was performed in a single institution with a low volume of patients with resectable PDAC, the restricted number of patients may have an impact on the multivariate analysis. Further well design, randomized studies are required.*

## Reviewers’ Comments to the Revised Manuscript

**Reviewer 1: anonymous**

Date received: 14-Feb-2022
Reviewer recommendation: **Accept in present form**
Reviewer overall scoring: **High**

Assessment Form scores: 5 = High/Yes; 3 = Medium/Adequate; 1 = Low

| | | | | | |
|---|---|---|---|---|---|
| Is the subject area appropriate for the journal | | 4 | | | |
| Does the title clearly reflect the paper's content? | | 4 | | | |
| Does the abstract clearly reflect the paper's content | | 4 | | | |
| Do the keywords clearly reflect the paper's content? | | 4 | | | |
| Does the introduction present the problem clearly? | | 4 | | | |
| Are the results/ conclusions justified? | | 4 | | | |
| How comprehensive and up-to-date is the subject matter presented? | | 4 | | | |
| How adequate is the data presentation? | | 4 | | | |
| Are units and terminology used correctly? | | 4 | | | |
| Is the number of cases adequate? | | 4 | | | |
| Are the experimental methods/ clinical studies adequate? | | 4 | | | |
| Is the length appropriate in relation to the content? | | 4 | | | |
| Does the reader get new insights from the article? | | 4 | | | |
| Please rate the practical significance. | | 4 | | | |
| Please rate the accuracy of methods. | | 4 | | | |
| Please rate the statistical evaluation and quality control. | | 4 | | | |
| Please rate the appropriateness of the figures and tables. | | 4 | | | |
| Please rate the appropriateness of the references. | | 4 | | | |
| Please evaluate the writing style and use of language. | | 4 | | | |
| Please judge the overall scientific quality of the manuscript. | | 4 | | | |
| Are the methods used worthy of reproduction in greater deal? | No | | | | |
| Would you be willing to review a revision of this manuscript? | Yes | | | | |

**Comments to author:** All queries were resolved. ..

## Comments by the Editor-in-Chief to Revised Submission

All reviewer comments were addressed adequately and the manuscript should be published in its present stage.
